# Supplementary material for: CNBP Binds and Unfolds In Vitro G-Quadruplexes Formed in the SARS-CoV-2 Positive and Negative Genome Strands
Source: Int J Mol Sci. 2021 Mar 5;22(5):2614. doi: 10.3390/ijms22052614 (PMC7961906; doi:10.3390/ijms22052614)
Supplement: Supplementary file 1 [file ijms-22-02614-s001.zip › Supplementary Table S3-Proofreading.docx]

**Table S3. Analysis of variations within selected PQSs in +gRNA and −gRNA of the SARS-CoV-2**

1. **Selected PQSs in +gRNA**

| **PQS**  **name** | **PQS/mutation**  **position** | **Sequence** | **Mutation Frequency/ Entropy** | **Protein** | **Codon** | **Amino acid change** | **G4RNA Screener** | | | **QGRS**  **mapper** | **PQS finder** |
| --- | --- | --- | --- | --- | --- | --- | --- | --- | --- | --- | --- |
|  |  |  |  |  |  |  | **cGcC** | **G4H** | **G4NN** |  |  |
| **+644** | 644 - 663 | **GG**UAAUAAA**GG**AGCU**GG**U**GG** |  | ORF1ab – nsp1 |  |  | 17 | 0.8 | 0.7006 | 30 | 20 |
|  | 649 | **GG**UAA**C**AAA**GG**AGCU**GG**U**GG** | <1% / 0.002 |  | 128 | N→ N | 8.5 | 0.75 | 0.5673 | 30 | 20 |
|  | 652 | **GG**UAAUAA**GGG**AGCU**GG**U**GG** | <1% / 0.004 |  | 129 | L→ L | 23 | 1.05 | 0.9064 | 31 | 20 |
|  | 655 | **GG**UAAUAAA**GGG**GCU**GG**U**GG** | <1% / 0.002 |  | 130 | G→ G | 32 | 1.35 | 0.9562 | 30 | 20 |
|  | 658 | **GG**UAAUAAA**GG**AGC**CGG**U**GG** | <1% / 0.006 |  | 131 | A→ A | 4.25 | 0.65 | 0.5295 | 30 | 20 |
| **+3,467** | 3,467 – 3,484 | **GG**A**GG**A**GG**UGUUGCA**GG**A |  | ORF1ab – nsp3 |  |  | 18 | 0.9444 | 0.8983 | 30 | 24 |
|  | 3,468 | **GA**A**GG**A**GG**UGUUGCA**GG**A  **GU**A**GG**A**GG**UGUUGCA**GG**A | <1% / 0.005  <1% / 0.005 |  | 1,068 | G→ E  G→ V | 15  15 | 0.7778  0.7778 | 0.899  0.8859 | -  - | -  - |
|  | 3,470 | **GG**A**AG**A**GG**UGUUGCA**GG**A | <1% / 0.004 |  | 1,069 | G→ R | 15 | 0.7778 | 0.6298 | - | - |
|  | 3,472 | **GG**A**GGGGG**UGUUGCA**GG**A | <1% / 0.008 |  | 1,069 | G→ G | 45 | 1.6111 | 0.9924 | 30 | 24 |
|  | 3,476 | **GG**A**GG**A**GG**UAUUGCA**GG**A | <1% / 0.002 |  | 1,071 | V→ I | 17 | 0.8889 | 0.0913 | 30 | 24 |
|  | 3,478 | **GG**A**GG**A**GG**UGUAGCA**GG**A | <1% / 0.002 |  | 1,071 | V→ V | 18 | 0.9444 | 0.8053 | 30 | 24 |
|  | 3,481 | **GG**A**GG**A**GG**UGUUGC**GGG**A | <1% / 0.002 |  | 1,072 | A→ A | 24 | 1.2222 | 0.9466 | 31 | 26 |
|  | 3,482 | **GG**A**GG**A**GG**UGUUGCA**AG**A | <1% / 0.013 |  | 1073 | G→ R | 15 | 0.7778 | 0.5889 | - | - |
|  | 3,483 | **GG**A**GG**A**GG**UGUUGCA**GA**A  **GG**A**GG**A**GG**UGUUGCA**GU**A | <1% / 0.018  <1% / 0.018 |  | 1,073 | G→ E  G→ V | 15  15 | 0.7778  0.7778 | 0.5889  0.7593 | -  - | -  - |
|  | 3,484 | **GG**A**GG**A**GG**UGUUGCA**GGG** | <1% / 0.012 |  | 1,073 | G→ G | 24 | 1.2222 | 0.945 | 30 | 24 |
| **+28,903** | 28,903 – 28,917 | **GG**CU**GG**CAAU**GG**C**GG** |  | N |  |  | 5.3333 | 0.8667 | 0.568 | 33 | 27 |
|  | 28,903 | **UG**CU**GG**CAAU**GG**C**GG**  **AG**CU**GG**CAAU**GG**C**GG** | <1% / 0.018  <1% / 0.018 |  | 210 | M→ I  M→ I | 4.3333  4.3333 | 0.6667  0.6667 | 0.4905  0.3956 | -  - | -  - |
|  |  | **CG**CU**GG**CAAU**GG**C**GG** | <1% / 0.018 |  |  | M→ I | 3.25 | 0.6 | 0.5032 | - | - |
|  | 28,904 | **GA**CU**GG**CAAU**GG**C**GG**  **GU**CU**GG**CAAU**GG**C**GG** | <1% / 0.009  <1% / 0.009 |  | 211 | A→ T  A→ S | 4.3333  4.3333 | 0.6667  0.6667 | 0.4959  0.4734 | -  - | -  - |
|  | 28,905 | **GGU**U**GG**CAAU**GG**C**GG** | <1% / 0.021 |  | 211 | A→ V | 8 | 0.9333 | 0.7033 | 33 | 27 |
|  | 28,907 | **GG**CU**UG**CAAU**GG**C**GG** | <1% / 0.012 |  | 212 | G→ C | 4.3333 | 0.6667 | 0.4437 | - | - |
|  | 28,908 | **GG**CU**GU**CAAU**GG**C**GG** | <1% / 0.002 |  | 212 | G→ V | 4.3333 | 0.6667 | 0.3143 | - | - |
|  | 28,910 | **GG**CU**GG**C**U**AU**GG**C**GG** | <1% / 0.002 |  | 213 | N→ Y | 5.3333 | 0.8667 | 0.0151 | 33 | 27 |
|  | 28,911 | **GG**CU**GG**CA**U**U**GG**C**GG** | <1% / 0.002 |  | 213 | N→ I | 5.3333 | 0.8667 | 0.3595 | 33 | 27 |
|  | 28,913 | **GG**CU**GG**CAAU**UG**C**GG** | <1% / 0.006 |  | 214 | G→ C | 4.3333 | 0.6667 | 0.2619 | - | - |
|  |  | **GG**CU**GG**CAAU**-G**C**GG** | <1% / 0.006 |  |  | Frameshift | 4.3333 | 0.7143 | 0.4865 | - | - |
|  | 28,914 | **GG**CU**GG**CAAU**GU**C**GG** | <1% / 0.002 |  | 214 | G→ V | 4.3333 | 0.6667 | 0.5144 | - | - |
|  |  | **GG**CU**GG**CAAU**G-**C**GG** | <1% / 0.002 |  |  | Frameshift | 4.3333 | 0.7143 | 0.4865 | - | - |
|  | 28,915 | **GG**CU**GG**CAAU**GGUGG** | <1% / 0.04 |  | 214 | G→ G | 8 | 0.9333 | 0.6745 | 33 | 27 |
|  |  | **GG**CU**GG**CAAU**GG-GG** | <1% / 0.04 |  |  | Frameshift | 14 | 1.5714 | 0.9608 | - | - |
|  | 28,916 | **GG**CU**GG**CAAU**GG**C**AG**  **GG**CU**GG**CAAU**GG**C**-G** | <1% / 0.008  <1% / 0.008 |  | 215 | G→ S  Frameshift | 4.3333  4.3333 | 0.6667  0.7143 | 0.58  0.6022 | -  - | -  - |
|  | 28,917 | **GG**CU**GG**CAAU**GG**C**GU**  **GG**CU**GG**CAAU**GG**C**G-** | <1% / 0.002  <1% / 0.002 |  | 215 | G→ V  Frameshift | 4.3333  4.3333 | 0.6667  0.7143 | 0.5739  0.6022 | -  - | -  - |

1. **Selected PQSs in -gRNA**

| **PQS**  **name** | **PQS/mutation**  **position**  **(+gRNA)** | **Sequence**  **(-gRNA)** | **Mutation Frequency/ Entropy** | **Protein**  **(+gRNA)** | **Codon**  **(+gRNA)** | **Amino acid change**  **(+gRNA)** | **G4RNA Screener** | | | **QGRS**  **mapper** | **PQS finder** |
| --- | --- | --- | --- | --- | --- | --- | --- | --- | --- | --- | --- |
|  |  |  |  |  |  |  | **cGcC** | **G4H** | **G4NN** |  |  |
| **-13,963**  **(position in +gRNA +15,941)** | 15,924 – 15,941 | **GG**AUCU**GGG**UAA**GG**AA**GG** |  | ORF1ab- nsp12 |  |  | 22 | 1.1111 | 0.9722 | 34 | 23 |
|  | 15,924 | **GG**AUCU**GGG**UAA**GG**AA**GA** | <1% / 0.006 |  | 819 | Y→ Y | 19 | 0.9444 | 0.868 | - | - |
|  | 15,925 | **GG**AUCU**GGG**UAA**GG**AA**AG** | <1% / 0.002 |  | 820 | L→ F | 19 | 0.9444 | 0.9351 | - | - |
|  | 15,927 | **GG**AUCU**GGG**UAA**GGG**A**GG** | <1% / 0.002 |  | 820 | L→ L | 28 | 1.3889 | 0.9859 | 34 | 23 |
|  | 15,936 | **GG**AUC**GGGG**UAA**GG**AA**GG** | <1% / 0.002 |  | 823 | P→ P | 32 | 1.5 | 0.9901 | 34 | 23 |
|  | 15,939 | **GGG**UCU**GGG**UAA**GG**AA**GG** | <1% / 0.01 |  | 824 | D→ D | 28 | 1.3889 | 0.9789 | 34 | 24 |
| **-23,877**  **(position in +gRNA +6,027)** | 6,011 – 6,027 | **GG**AUAU**GG**UU**GG**UUU**GG** |  | ORF1ab – nsp3 |  |  | 160 | 0.9412 | 0.0217 | 34 | 24 |
|  | 6,011 | **GG**AUAU**GG**UU**GG**UUU**GA** | <1% / 0.006 |  | 1,916 | P→ S | 130 | 0.7647 | 0.0182 | - | - |
|  | 6,016 | **GG**AUAU**GG**UU**GA**UUU**GG** | <1% / 0.006 |  | 1,917 | Q→ * | 130 | 0.7647 | 0.0066 | - | - |
|  | 6,019 | **GG**AUAU**GGC**U**GG**UUU**GG** | <1% / 0.008 |  | 1,918 | Q→ Q | 16 | 0.8824 | 0.0141 | 34 | 24 |
|  | 6,022 | **GG**AUA**CGG**UU**GG**UUU**GG** | <1% / 0.004 |  | 1,919 | P→ P | 16 | 0.8824 | 0.3629 | 34 | 24 |
|  | 6,026 | **GA**AUAU**GG**UU**GG**UUU**GG** | <1% / 0.018 |  | 1,921 | P→ S | 130 | 0.7647 | 0.01 | - | - |
|  | 6,027 | **AG**AUAU**GG**UU**GG**UUU**GG** | <1% / 0.034 |  | 1,921 | P→ L | 130 | 0.7647 | 0.0041 | - | - |

**Color references:**

**Mutations that disrupt G-tracts and may lead to impede PQSs or reduce PQSs scores are shown in red**

**Mutations that do not disturb G-tracts and may be neutral for PQSs are shown in yellow**

**Mutations that produce G-tracts extension and may increase PQSs scores are shown in green**

**Dashes indicate single nucleotide deletions and are highlighted with the same color code as substitutions in respect to their effect on PQSs.**

**Synonymous or silent mutations are shaded in light blue in the amino acid change column**

**Not synonymous or missense mutations are shaded in light pink in the amino acid change column**

**Nonsense mutations (leading to stop codons) are highlighted with red shade in the amino acid change column**

**Frameshift mutations are highlighted with yellow shade in the amino acid change column**

**Scores overpassing the defined threshold (G4RNA Screener)**
