# Supplementary material for: CNBP Binds and Unfolds In Vitro G-Quadruplexes Formed in the SARS-CoV-2 Positive and Negative Genome Strands
Source: Int J Mol Sci. 2021 Mar 5;22(5):2614. doi: 10.3390/ijms22052614 (PMC7961906; doi:10.3390/ijms22052614)
Supplement: Supplementary file 1 [file ijms-22-02614-s001.zip › Supplementary Table S4-Proofreading.docx]

**Table S4. Prediction of secondary structures and minimum free energy (MFE) of the selected PQSs in +gRNA and −gRNA of the SARS-CoV-2.**

**Parameters for RNAfold and NUPACK were set as the default values, setting temperature at 20 °C or 37 °C and selecting to incorporate G–Quadruplex formation into the RNAfold structure prediction algorithm.**

1. **Selected PQSs in +gRNA**

| **PQS** | **RNAfold**  **Base-pair probability**  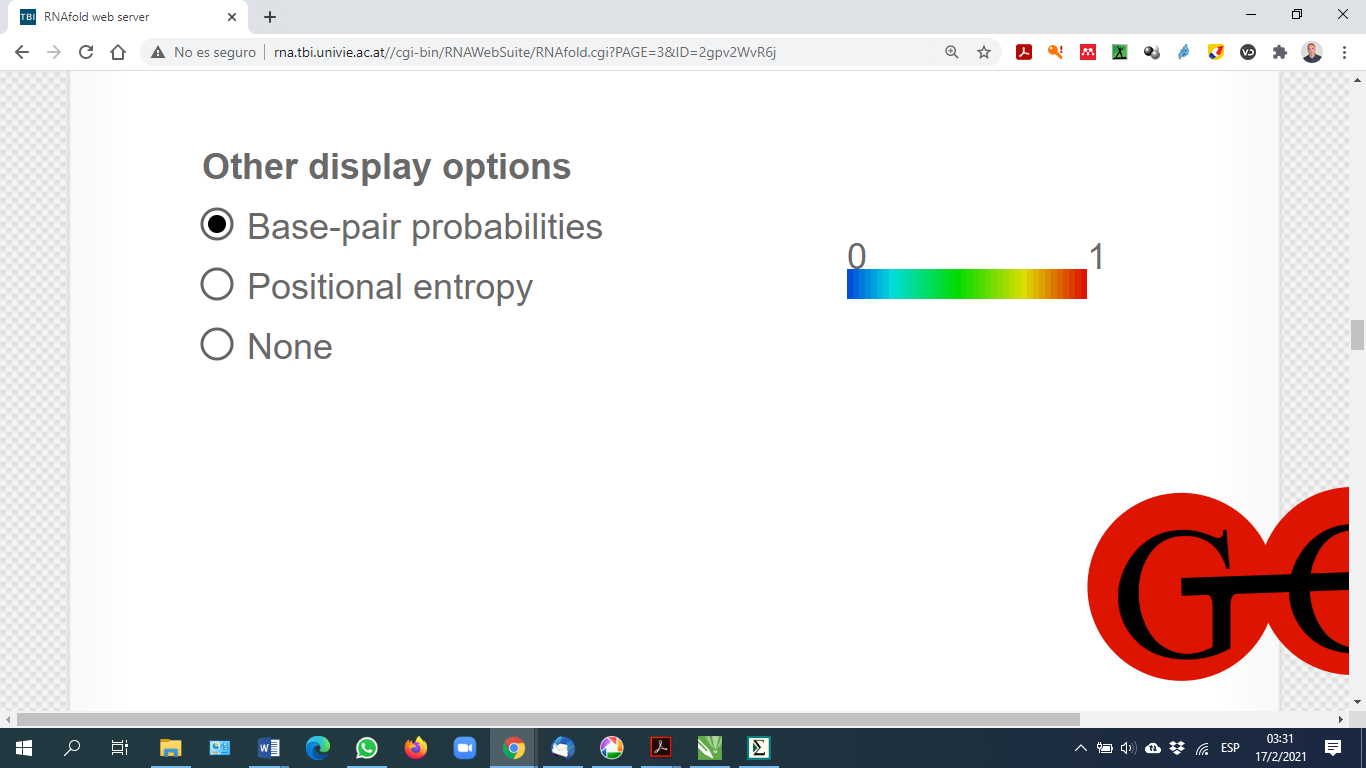 | | **NUPACK**  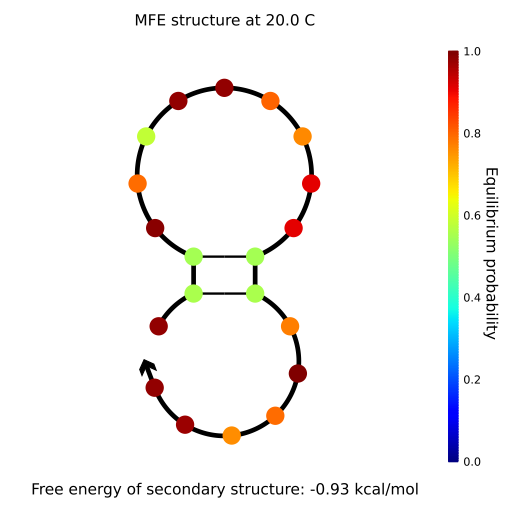 | |
| --- | --- | --- | --- | --- |
|  | **MFE at 20 °C (kcal/mol)** | **MFE at 37 °C (kcal/mol)** | **MFE at 20 °C (kcal/mol)** | **MFE at 37 °C (kcal/mol)** |
| **+644** | 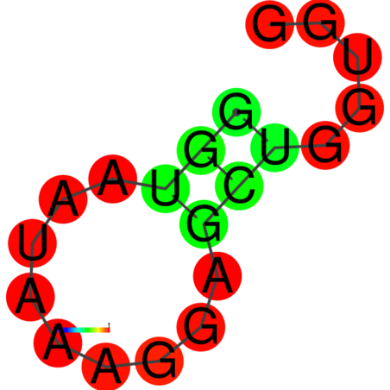 | 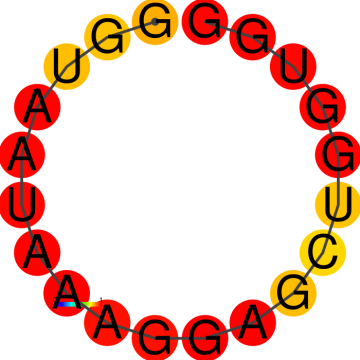 | 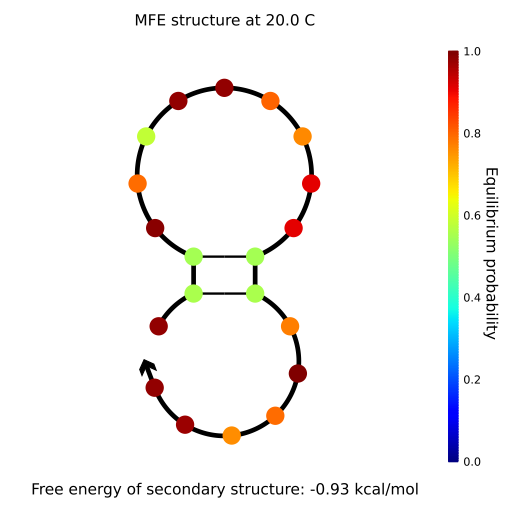 | 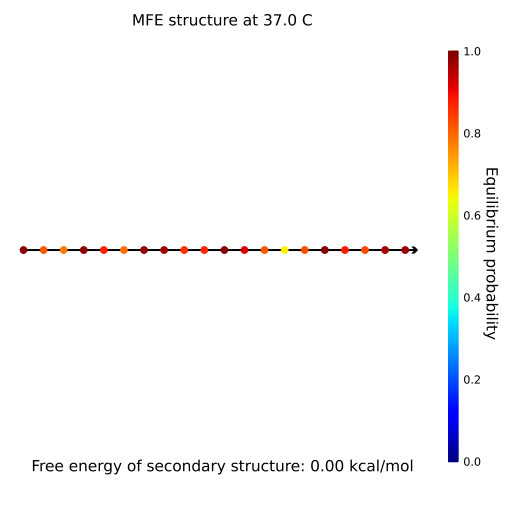 |
|  | **−0.20** | **00** | −**0.93** | **00** |
| **+3,467** | 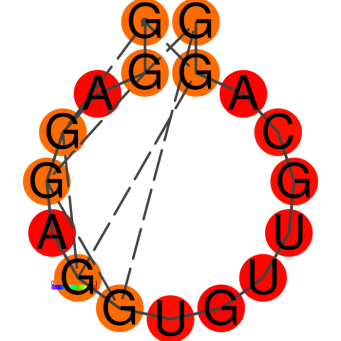 | 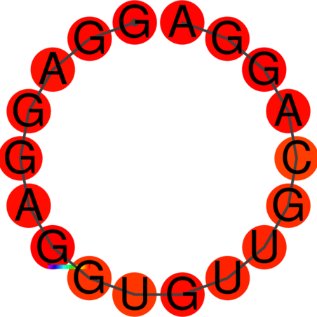 | 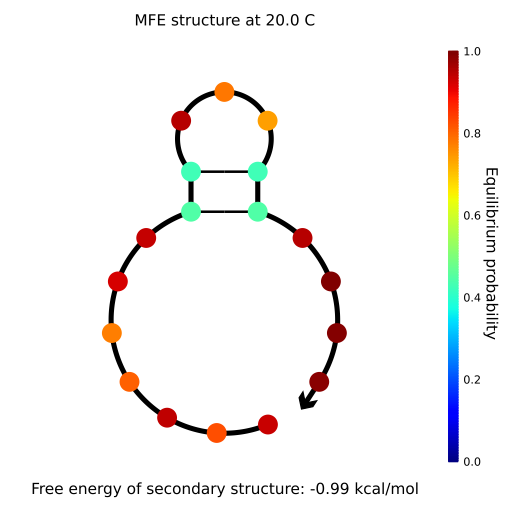 | 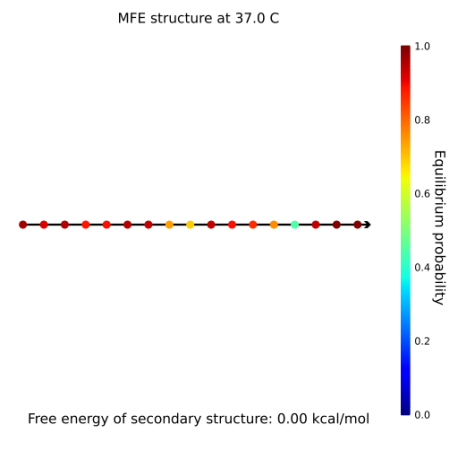 |
|  | **−1.48** | **00** | **−0.99** | **00** |
| **+28,903** | 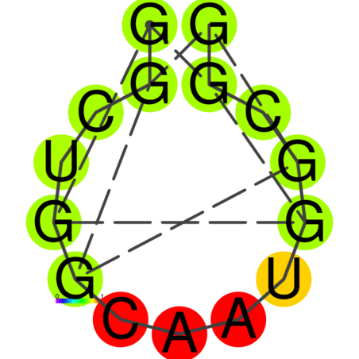 | **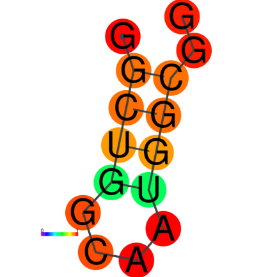** | 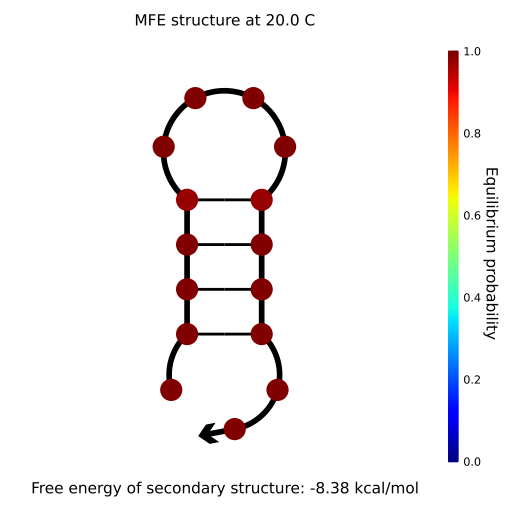 | 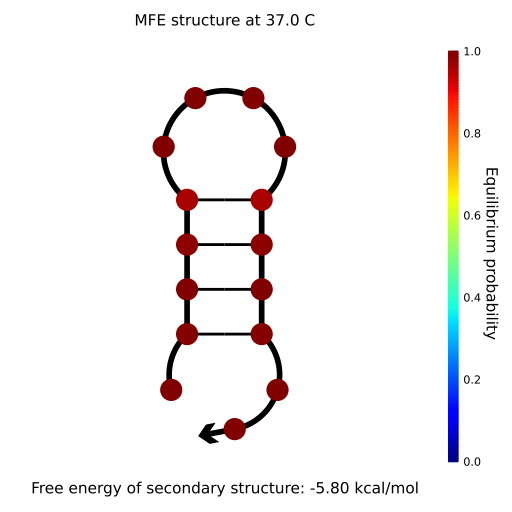 |
|  | **−5.30** | **−2.20** | **−8.38** | **−5.80** |

1. **Selected PQSs in −gRNA**

| **PQS** | **RNAfold**  **Base-pair probability**  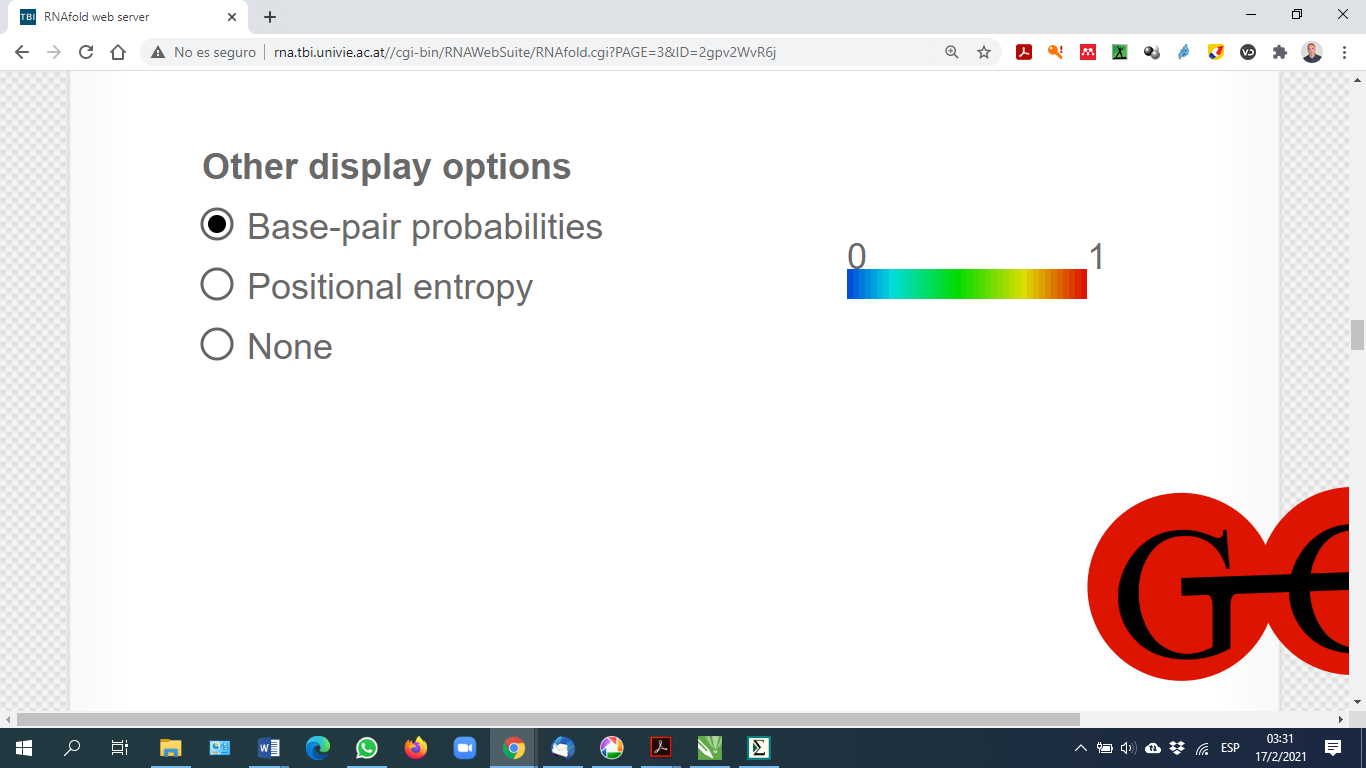 | | **NUPACK**  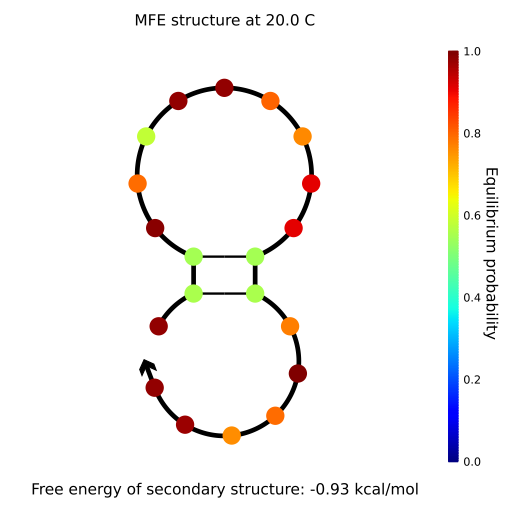 | |
| --- | --- | --- | --- | --- |
|  | **MFE at 20 °C (kcal/mol)** | **MFE at 37 °C (kcal/mol)** | **MFE at 20 °C (kcal/mol)** | **MFE at 37 °C (kcal/mol)** |
| **−13,963** | 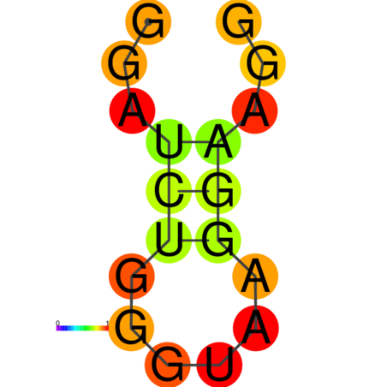 | 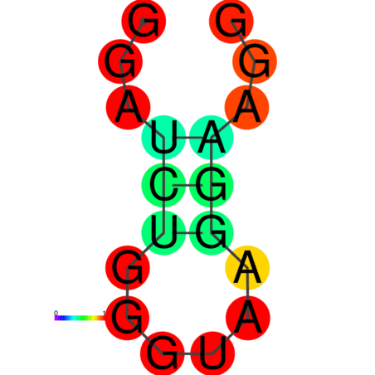 | 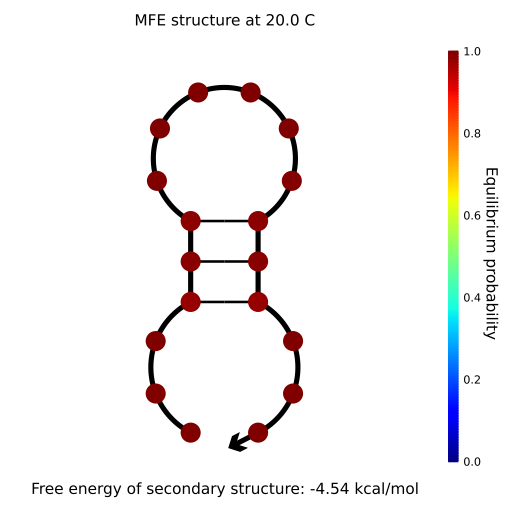 | 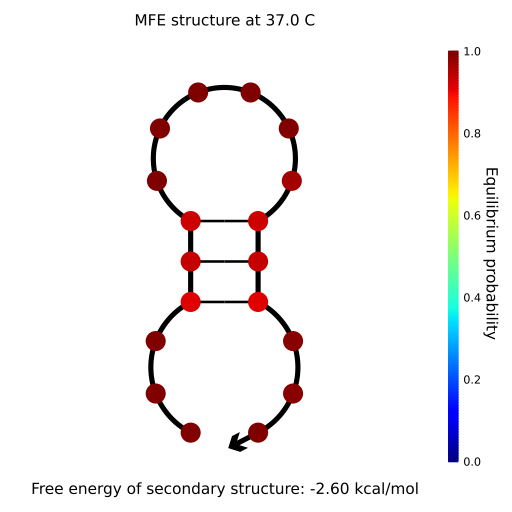 |
|  | −1.72 | −0.30 | −4.54 | −2.70 |
| **−23,877** | 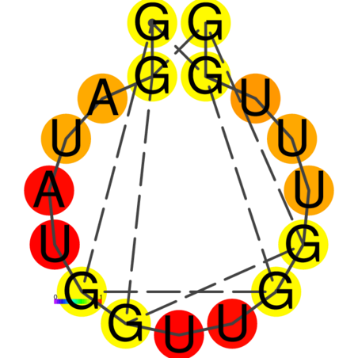 | 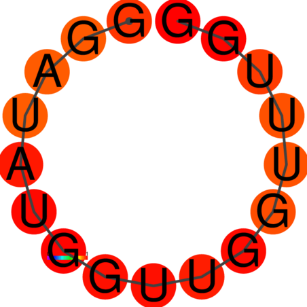 | 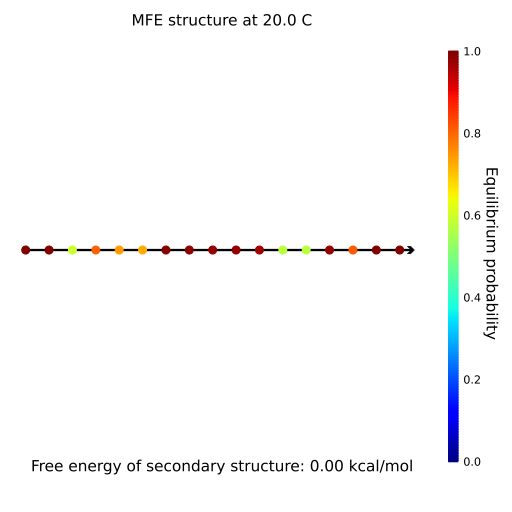 | 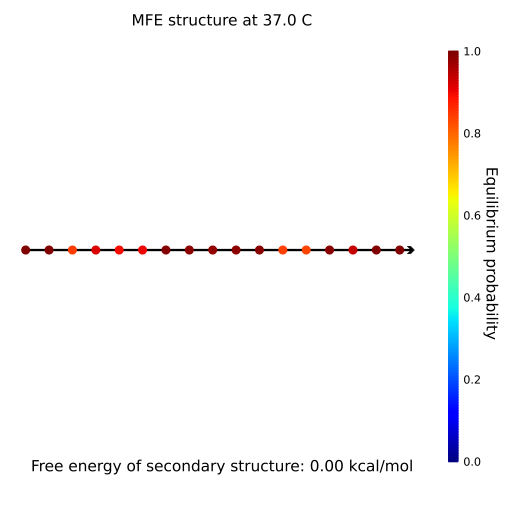 |
|  | −1.48 | 00 | 00 | 00 |
