# Supplementary material for: CNBP Binds and Unfolds In Vitro G-Quadruplexes Formed in the SARS-CoV-2 Positive and Negative Genome Strands
Source: Int J Mol Sci. 2021 Mar 5;22(5):2614. doi: 10.3390/ijms22052614 (PMC7961906; doi:10.3390/ijms22052614)
Supplement: Supplementary file 1 [file ijms-22-02614-s001.zip › Supplementary Figures-Proofreading.pdf]

Figure S1

(a)

Selected PQSs in +gRNA

		Location +gRNA	
SARS-CoV-2	GGUAAUAAAGGAGCUGGUGG	644	
RaTG13	GGUAAUAAAGGAGCUGGUGG	644	100% identity
Bat_C21	GGUAAUAAAGGAGCUGGUGG	643	100% identity
Bat_C45	GGUAAUAAAGGAGCUGGUGG	643	100% identity
SARS-CoV	GGUAAUAAGGGAGCCGGUGG	643	90% identity

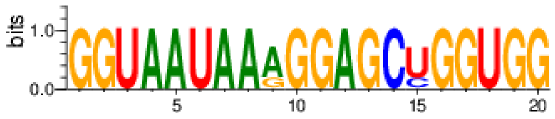

		Location +gRNA	
SARS-CoV-2	GGAGGAGGUGUUGCAGG	3,467	
RaTG13	GGAGGAGGUGUUGC-GG	3,464	94% identity
Bat_C21	GGAGGAGGUGUUGCUGG	3,385	94% identity
Bat_C45	GGAGGAGGUGUUGCUGG	3,451	94% identity

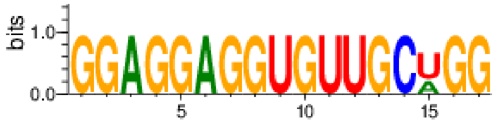

		Location +gRNA	
SARS-CoV-2	GGCUGGCAAUGGCGG-------	28,903	
RaTG13	GGAUGGC--UGGCAAUGG	28,880	66% identity
Bat_C21	GGCUGGCAAUGGCGG-------	28,739	100% identity
Bat_C45	GGCUGGCAAUGGCGG-------	28,808	100% identity
SARS-CoV	GGCUAGCGGAGGUGG-------	28,752	67% identity
MERS-CoV-2	---GGGUAUUGGCGGAGACAGG	28,791	60% identity

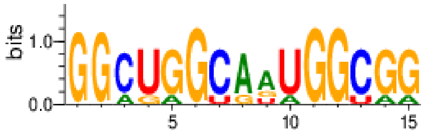

Figure S1

(b)

Selected PQSs in −gRNA

		Location −gRNA	Location +gRNA	
SARS-CoV-2	GGAUAUGGUUGGUUUGG	23,877	6,027	
RaTG13	GGAUAAGGUUGGUUUGG	23,832	6,024	100% identity
Bat_C21	GG-UAAGGUUGGUUUGG	23,786	5,947	88% identity
Bat_C45	GG-UAAGGUUGGUUUGG	23,790	6,013	88% identity

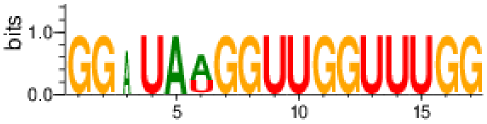

		Location −gRNA	Location +gRNA	
SARS-CoV-2	GGUGAUAGAGGUUUGUGGUGGUUGG------	19,865	10,039	
RaTG13	GGUGAUAGAGGUUUGUGGUGG----------	19,820	10,036	84% identity
Bat_C21	----------------GGUGGUUGGUAGAGG	19,789	9,944	36% identity
Bat_C45	----------------GGUGGUUGGUAGAGG	19,793	10,010	36% identity
MERS-CoV	GGUUAUGCUACAGUUGGGUGGUUGG------	20,116	10,004	60% identity

		Location −gRNA	Location +gRNA	
SARS-CoV-2	GGAUCUGGGUAAGGAAGG	13,963	15,941	
RaRG13	GGAUCCGGGUAAGGGAGG	13,918	15,938	89% identity
Bat_C21	GGAUCUGGAUAAGGCAGG	13,871	15,862	89% identity
Bat_C45	GGAUCUGGAUAAGGCAGG	13,875	15,928	89% identity
SARS-CoV	GGAUCUGGGUAAGGCAGG	13,881	15,871	94% identity
MERS-CoV	GG-UCUGGAUAAGGAAGG	14,209	15,911	89% identity

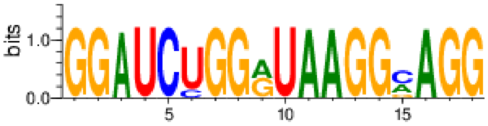

Figure S1

(c)

Not selected PQS

		Location +gRNA	
SARS-CoV-2	**GG**UAUGU**GG**AAA**GG**UUAU**GG**	13,385	
RaTG13	**GG**UAUGU**GG**AAA**GG**UUAU**GG**	13,382	100% identity
Bat_C21	**GG**AAUGU**GG**AAA**GG**UUAU**GG**	13,306	95% identity
Bat_C45	**GG**AAUGU**GG**AAA**GG**UUAU**GG**	13,372	95% identity
SARS-CoV	**GG**AAUGU**GG**AAA**GG**UUAU**GG**	13,315	95% identity

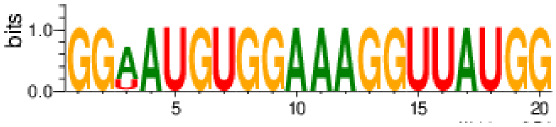

		Location −gRNA	Location +gRNA	
SARS-CoV-2	**GG**UUAAGU**GG**U**GG**UCUA**GG**	13,134	16,768	
RaRG13	**GG**UUAAGU**GG**U**GG**UCUA**GG**	13,091	16,765	100% identity

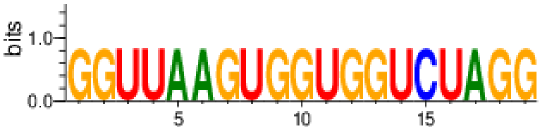

Figure S1. Multiple sequence alignments of the position-conserved selected SARS-CoV-2 PQSs identified in this study with their homologous PQSs in other members of the *Coronaviridae* family**.** (**a**) Selected PQSs in the +gRNA. (**b**) Selected PQSs in the −gRNA. (**c**) Not selected PQSs +13,385 of the +gRNA of SARS-CoV-2 (conserved in position and with significantly high cGcC scores, > 150) and −13,134 of the −gRNA of SARS-CoV-2 (conserved in sequence and position only in two viral genomes).

Figure S2

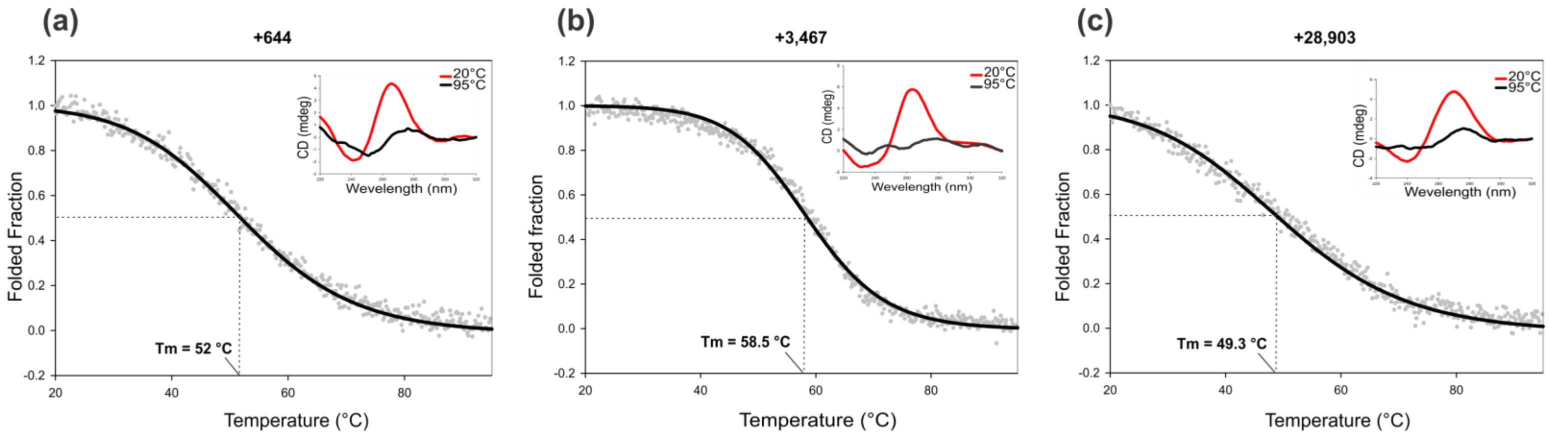

(a)

Tm (°C)	ΔH (cal/mol)	$\Delta G_{37\,°C}$ (cal/mol)
52	−21,524.4	−993.4

(b)

Tm (°C)	ΔH (cal/mol)	$\Delta G_{37\,°C}$ (cal/mol)
58.5	−32,270.7	−21,06.6

(c)

Tm (°C)	ΔH (cal/mol)	$\Delta G_{37\,°C}$ (cal/mol)
49.3	−18,331.1	−707.7

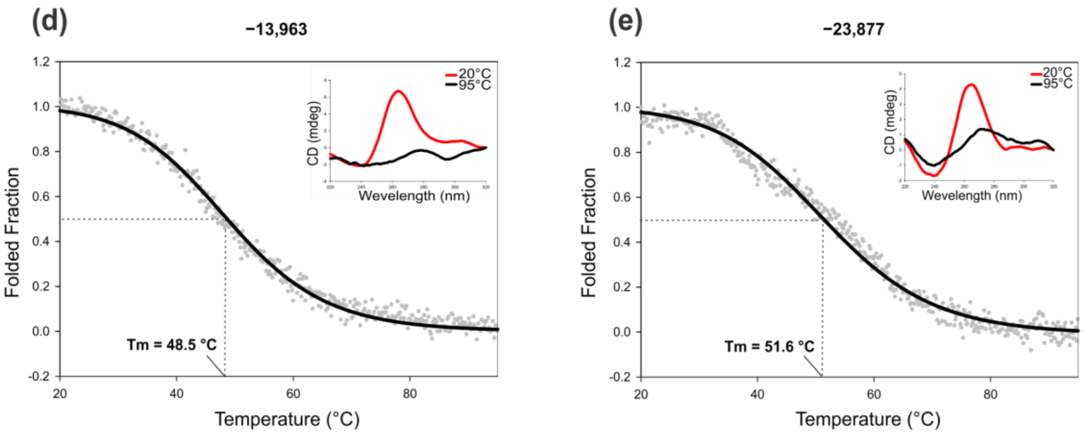

(d)

Tm (°C)	ΔH (cal/mol)	$\Delta G_{37\,°C}$ (cal/mol)
48.5	−24,016.6	−869.8

(e)

Tm (°C)	ΔH (cal/mol)	$\Delta G_{37\,°C}$ (cal/mol)
51.6	−22,431.8	−1,022

Figure S2. CD melting curves obtained for selected PQSs in the positive and negative-sense RNA genomes of SARS-CoV-2. (**a**), (**b**) and (**c**) show the results of selected PQSs in +gRNA of SARS-CoV-2: +644, +3,467 and +28,903, respectively. (**d**) and (**e**) show the results of selected PQSs in −gRNA of SARS-CoV-2: −13,963 and −23,877, respectively. Experimental data (dots) and fitted curves (solid lines) are represented. K^+ concentrations were 100 mM (+3,467 and −23,877) or 200 mM (+644, +28,903 and −13,963). Estimated melting temperatures (Tm), changes of enthalpies (ΔH) and change of free Gibbs energy at 37 °C ($\Delta G_{37\,°C}$) are informed below each graph. Insets in the top-right corners show CD spectra performed at the initial temperature (20 °C, red) and at the final temperature (95 °C, black) of the melting curve for each PQS.

Figure S3

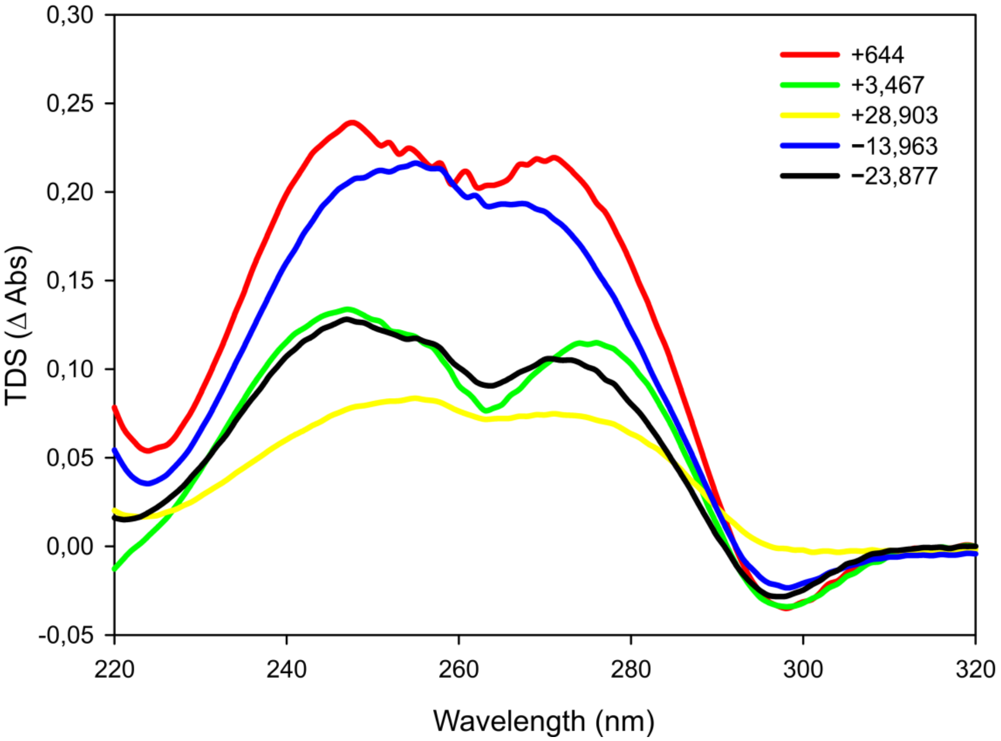

Figure S3. TDS obtained for selected PQSs in the positive and negative-sense RNA genomes of SARS-CoV-2. TDS resulting from the subtraction of the 20 °C absorbance spectra from the 70 °C absorbance spectra. K^+ concentrations were 100 mM (+3,467 and −23,877) or 200 mM (+644, +28,903 and −13,963).

Figure S4

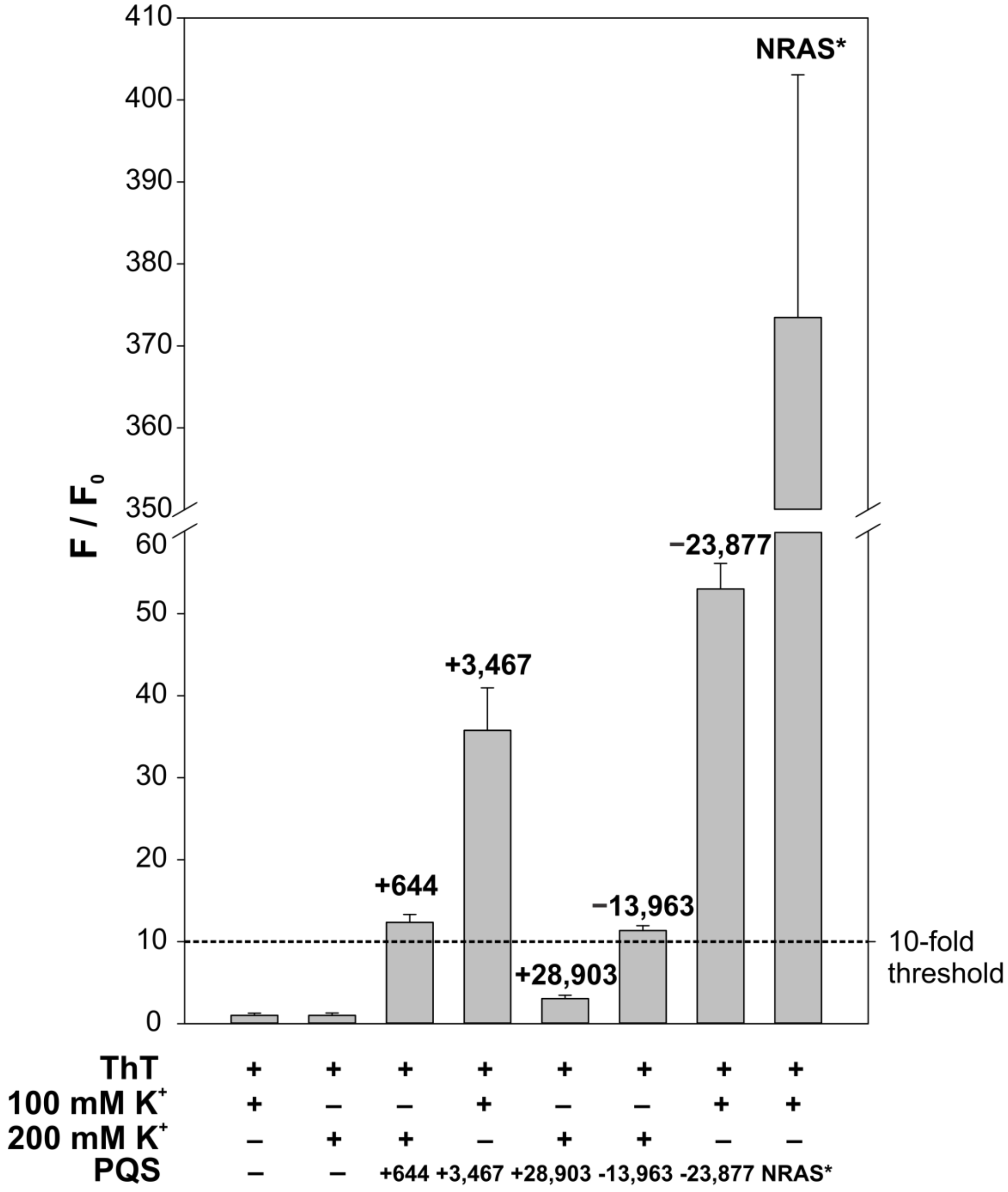

***NRAS** 5'-<u>**GGG**</u>A<u>**GGGG**</u>C<u>**GGG**</u>UCU<u>**GGG**</u>-3'

Figure S4. ThT fluorescence assay for selected PQSs in the positive and negative-sense RNA genomes of SARS-CoV-2. Bar graph of fluorescence enhancement (F/F_0) of ThT in the presence of the selected PQSs folded in the presence of 100 or 200 mM K^+ as indicated. Each bar represents the mean of three technical repeats and error bars correspond to standard deviation (SD). The marked threshold of 10-fold was used for considering G4 formation. Below the graph the sequence of NRAS RNA oligonucleotide used as positive control for the assay is represented.
